# Salvianolic acid B, a novel autophagy inducer, exerts antitumor activity as a single agent in colorectal cancer cells

**DOI:** 10.18632/oncotarget.11385

**Published:** 2016-08-19

**Authors:** Zhao Jing, Weiqiang Fei, Jichun Zhou, Lumin Zhang, Liuxi Chen, Xiaomin Zhang, Xiao Liang, Jiansheng Xie, Yong Fang, Xinbing Sui, Weidong Han, Hongming Pan

**Affiliations:** ^1^ Department of Medical Oncology, Sir Run Run Shaw Hospital, College of Medicine, Zhejiang University, Hangzhou, Zhejiang, China; ^2^ Biomedical Research Center and Key Laboratory of Biotherapy of Zhejiang Province, College of Medicine, Zhejiang University, Hangzhou, Zhejiang, China; ^3^ Department of Surgical Oncology, Sir Run Run Shaw Hospital, College of Medicine, Zhejiang University, Hangzhou, Zhejiang, China; ^4^ Department of General Surgery, Sir Run Run Shaw Hospital, College of Medicine, Zhejiang University, Hangzhou, Zhejiang, China

**Keywords:** salvianolic acid B, natural compound, autophagy, cell death, colorectal cancer

## Abstract

Salvianolic Acid B (Sal B), an active compound extracted from the Chinese herb *Salvia miltiorrhiza*, is attracting more and more attention due to its biological activities, including antioxidant, anticoagulant and antitumor effects. However, autophagy induction in cancer cells by Sal B has never been recognized. In this study, we demonstrated that Sal B induced cell death and triggered autophagy in HCT116 and HT29 cells in a dose-dependent manner. Specific inhibition of autophagy by 3-MA or shRNA targeting Atg5 rescued Sal B-induced cell death *in vitro* and *in vivo*, suggesting that Sal B-induced autophagy may play a pro-death role and contribute to the cell death of colorectal cancer cell lines. Furthermore, AKT/mTOR signaling pathway was demonstrated to be a critical mediator in regulating Sal B-induced cell death. Overexpression of AKT by the transfection with AKT plasmid or pretreatment with insulin decreased Sal B-induced autophagy and cell death. Inversely, inhibition of AKT by LY294002 treatment markedly enhanced Sal B-induced autophagy and cell death. Taken together, our results demonstrate, for the first time, that Sal B is a novel autophagy inducer and exerts its antitumor activity as a single agent in colorectal cancer cells through the suppression of AKT/mTOR pathway.

## INTRODUCTION

Colorectal cancer (CRC) is still the third most common cancer and the third leading cause of cancer-related death around the world, although considerable progress has been made in the treatment of CRC over the past years [1]. Currently, the initial chemotherapy with 5-fluorouracil (5-FU) and its derivatives is effective in most CRC patients, the relapse rate remains high due to increasing resistance to chemotherapeutic agents [2, 3]. Therefore, identifying novel compounds and overcoming chemoresistance will be a key issue for developing more effective individualized therapeutic strategies for the treatment of CRC patients.

Macroautophagy (hereafter referred to as autophagy) is an evolutionarily conserved catabolic process by which damaged or dysfunctional cellular contents are delivered to lysosomes for bulk degradation [4]. Autophagy is regulated by various signaling pathways, and AMP-activated protein kinase (AMPK) and mammalian target of rapamycin (mTOR) signaling pathways have emerged as the central checkpoints in the regulation of autophagy [5]. The role of autophagy in regulating cancer cell fate remains controversial. Autophagy itself fulfils a dual role, having pro-survival and pro-death properties depending on tumor types and treatment characteristic [6]. In response to anticancer treatments, autophagy can be activated as a protective mechanism to mediate the acquired resistance to anticancer therapies. Paradoxically, on the other hand, autophagy may also function as a death executioner to induce autophagic cell death, a distinct form of cell death in contrast to type I programmed cell death or apoptosis [7–9].

In the past few years, natural products with anticancer activity have gained more and more attention due to their favorable safety and efficacy profiles. *Salvia miltiorrhiza* (Danshen), a well-known traditional Chinese medical herb, has been widely and successfully used for treating cardio- and cerebral vascular diseases, such as angina pectoris, myocardial infarction (MI) and stroke [10]. Salvianolic Acid B (Sal B), one of the major water-soluble compounds of Danshen, is the most abundant and bioactive component of the salvianolic acids in Danshen [10]. Previous studies have shown that Sal B possesses multiple desirable potentials, including anti-oxidation, anti-inflammation, anti-coagulation and indirect regulation of immune function [11–13]. Recent studies indicate that Sal B also exerts anticancer effects in several cancer cell lines including glioma, hepatoma, oral squamous carcinoma, as well as head and neck squamous cell cancers [14–17]. However, the effect of Sal B on autophagy and the survival of CRC cells has never been reported.

In the present study, we investigated the effect of Sal B on CRC cells. We demonstrated, for the first time, that Sal B was a novel autophagy inducer, with significant antitumor efficacy as a single agent by inducing autophagic cell death in CRC cells. Furthermore, we showed that AKT inhibition is a key determinant for Sal B-mediated autophagic cell death. To the best of our knowledge, this is the first research to demonstrate that Sal B induces autophagic cell death through the AKT-mTOR signaling in human CRC cells. Our results suggest that Sal B may be an attractive therapeutic strategy for the treatment of colorectal cancer.

## RESULTS

### Sal B induces cell death and inhibits cell proliferation in CRC cell lines

**Figure 1:**
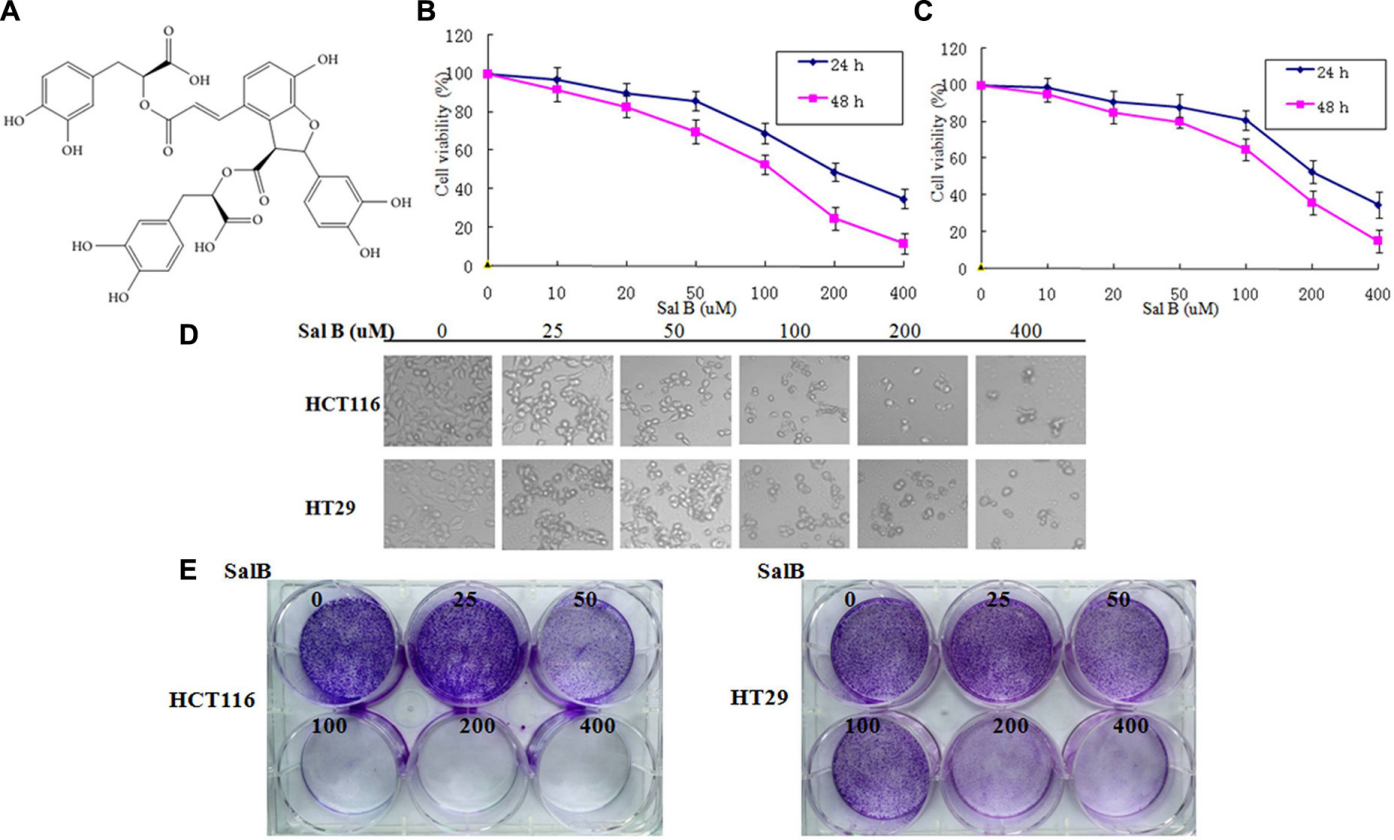
The effect of sal B on cell viability and proliferation in CRC cell lines (**A**) The chemical structures of Sal B. (**B**) The cell viability of HCT116 cells was measured via the MTT assay after Sal B treatment. The experiments were performed in triplicate. (**C**) The cell viability of HT29 cells was measured via the MTT assay after Sal B treatment. The experiments were performed in triplicate. (**D**) Representative cell morphological changes are detected by light microscopy; characteristic morphological features of cell death were observed, including detachment and cell shrinkage. (**E**) Representative colony formation assay by monolayer culture.

In order to examine whether Sal B (Figure 1A) affects human colorectal cancer cell growth, we first investigated the effect of Sal B on cell viability in HCT116 and HT29 cells. After treatment with different concentrations of Sal B for 24 and 48 h, Sal B significantly inhibited the growth of CRC cells in a dose- and time-dependent manner (Figure 1B and 1C). Next, we used various concentrations of Sal B in the treatment of HCT116 and HT29 cells for 24 h in subsequent experiments. Light microscopy showed that the viability of HCT116 and HT29 cells treated with Sal B was significantly lower than that of controls (Figure 1D), with more detached and shrunken cells appearing. To determine whether Sal B inhibits anchorage-independent growth, we performed colony formation assays through monolayer culture. In agreement with MTT viability assay results, Sal B remarkably decreased the number and the size of the colonies (Figure 1E). These results suggest that Sal B possesses growth-inhibitory potential in CRC cells as a single agent.

### Sal B triggers autophagy in CRC cell lines

**Figure 2:**
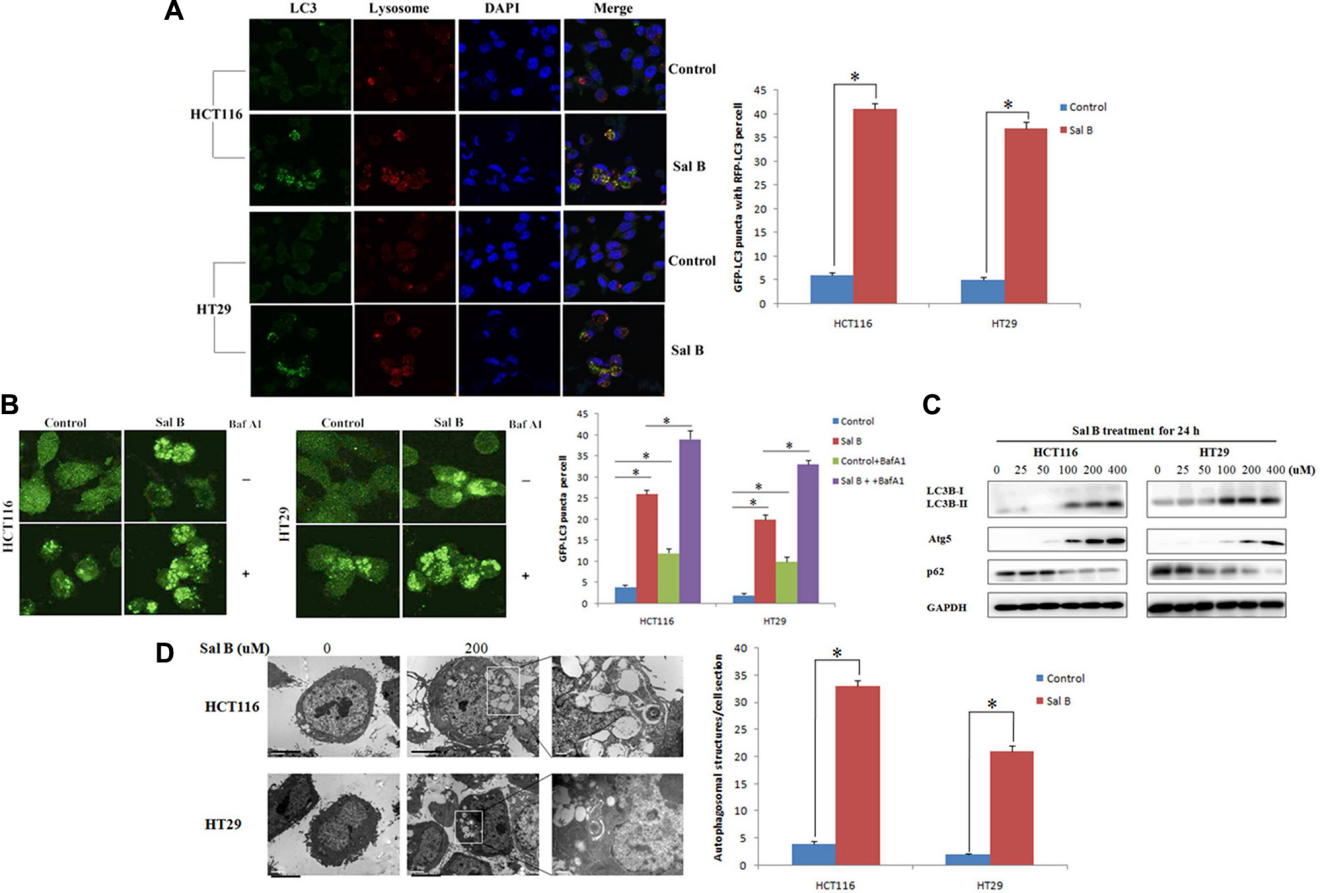
Aberrant autophagosome accumulation is involved in sal B-treated cells (**A**) Cells were treated with Sal B for 24 h before they were labeled with a fluorescent marker and imaged by fluorescence microscopy. Green: FITC-labeled LC3; Red: lyso-tracker-labeled lysosome; Blue: DAPI-labeled nucleus. *p < 0.05. (**B**) GFP-LC3 stable cell lines were made and Sal B-induced autophagic flux was detected in the presence and absence of BafA1 (**C**) The conversion from LC3-I to LC3-II as well as Atg 5 and p62, was detected by western blotting. (**D**) Electron microscopy shows ultrastructures of autophagosome in these cells. The experiments were performed in triplicate. Bar = 1 μm. *p < 0.05.

To investigated whether autophagy occurred in Sal B-treated cells, we examined the effect of Sal B on autophagy. After HCT116 and HT29 cells were treated with Sal B for 24 h, we performed fluorescence assays for LC3B to validate the effects of Sal B on autophagy. As a result, specific punctate distribution of endogenous LC3-II was observed in Sal B-treated cells and the percentage of FITC–LC3 positive cells with punctate staining significantly increased in Sal B-treated cells, compared with their controls (Figure 2A). In addition, treatment of Sal B to stable CRC cell lines expressing GFP-tagged LC3 resulted in marked accumulation of green fluorescent dots than untreated controls, suggesting induction of autophagy (Figure 2B). Sal B-induced autophagic flux was further investigated in the presence and absence of autophagosome– lysosome fusion inhibitors, bafilomycin A1 (BafA1). HCT116 and HT29 cells were preincubated with 100 nM BafA1 for 2 h and then treated with Sal B for 24 h. As a result, enhanced accumulation of LC3 puncta was found after 24 h treatment of Sal B in cells pre-incubated with BafA1 (Figure 2B). We next performed western blotting analysis to detect cleaved LC3-II and found that a significantly increased LC3-II/I ratio was shown in HCT116 and HT29 cells treated with Sal B for 24 h (Figure 2C). At last, transmission electron microscopy was used to further confirm the morphological changes in Sal B-treated cells. As shown in Figure 2D, most of the HCT116 and HT29 cells with Sal B treatment displayed an extensive accumulation of double or multimembraned structures with a broad range of morphologies, indicating the formation of autophagosomes. These results suggest that aberrant autophagosome accumulation is involved in Sal B-treated cells.

### Sal B-induced cell death is partially apoptotic

**Figure 3:**
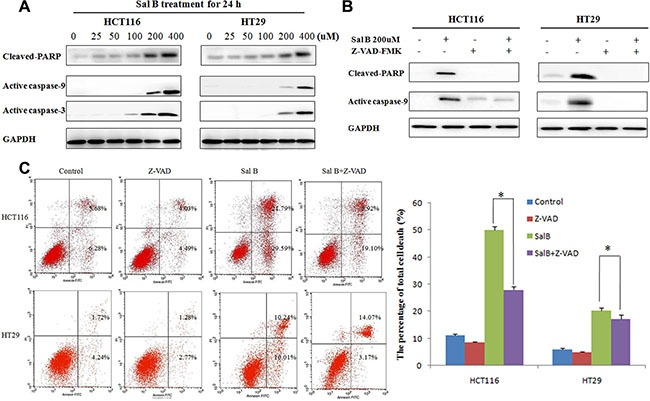
Sal B-induced cell death is partially apoptotic (**A**) The expression of apoptosis-associated protein cleaved PARP, active caspase-9 and caspase-3 in cells treated with Sal B for 24 h. (**B**) The expression of cleaved PARP and active caspase-9 were detected by western blotting after the cells were treated with Sal B in the presence or absence of Z-VAD-FMK. (**C**) Flow cytometric analysis of cell death. The data are shown as the mean ± SD. The experiments were performed in triplicate. *p < 0.05.

Next, we investigated the role of apoptosis in Sal B-induced cell death. Western blotting analysis showed that the cleaved caspase-9, caspase-3 and poly (ADP-ribose) polymerase (PARP) were increased in HCT116 and HT29 cells treated with Sal B for 24 h (Figure 3A), indicating that apoptosis may be involved in Sal B-induced cell death. To further elucidate whether caspase activation is required for Sal B-induced cell death, we exposed cells to a pan caspase inhibitor Z-VAD-FMK. As shown in Figure 3B, the addition of Z-VAD-FMK (25 μM) markedly suppressed the exoression of cleaved PARP1 and active caspase-9 induced by Sal B (Figure 3B). However, Sal B-induced cell death was only modestly rescued in all the treated CRC cell lines (Figure 3C), which was confirmed by an Annexin V–FITC dual staining assay followed by flow cytometry. Taken together, our data demonstrate that Sal B induced cell death in a partially apoptotic manner.

### Autophagy contributes to sal B-induced cell death

**Figure 4:**
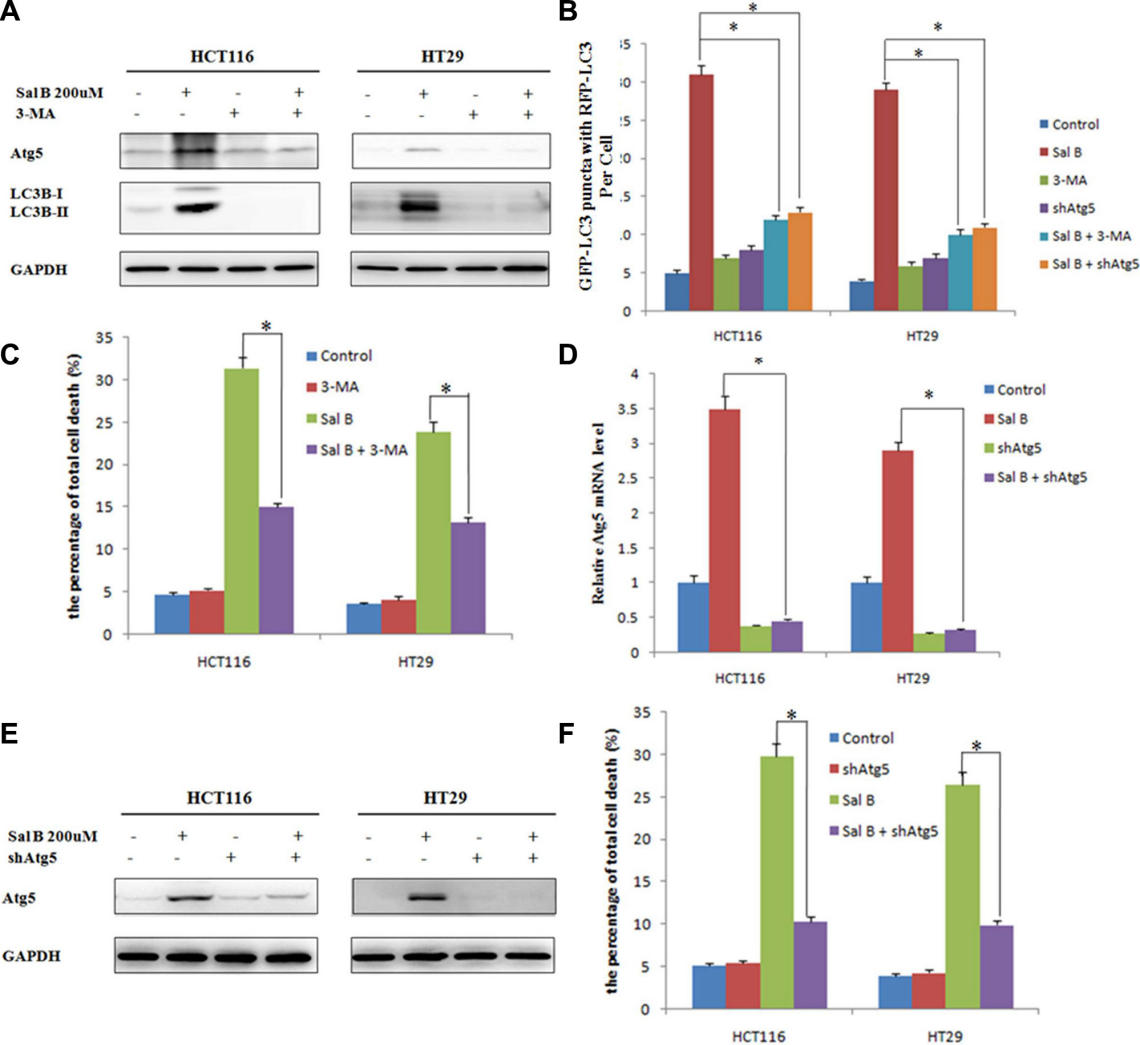
Autophagy contributes to sal B-induced cell death (**A**) The expression of Atg5 and LC3-II/I ratio were detected by western blotting after the cells were treated with Sal B in the presence or absence of 3-MA. (B) Cells were treated with Sal B in the presence or absence of 3-MA for 24 h before they were labeled with a fluorescent marker and imaged by fluorescence microscopy. Green: FITC-labeled LC3; Red: lyso-tracker-labeled lysosome; Blue: DAPI-labeled nucleus. *p < 0.05. (**C**) Flow cytometric analysis of cell death. The data are shown as the mean ± SD. The experiments were performed in triplicate. *p < 0.05. (**D**) The Atg5 mRNA level was detected by RT-PCR after the cells were treated with Sal B in the presence or absence of shAtg5. (**E**) The protein expression of Atg5 was detected by western blotting after the cells were treated with Sal B in the presence or absence of shAtg5. (**F**) Flow cytometric analysis of cell death. The data are shown as the mean ± SD. The experiments were performed in triplicate. *p < 0.05.

To assess whether autophagy contributed to Sal B-induced cell death in HCT116 and HT29 cells, we first treated these cells with the autophagy inhibitor 3-methyladenine (3-MA). 3-MA, a class III phosphatidylinositol 3-kinase (PtdIns3K) inhibitor, can block autophagosome formation. The combination of Sal B with 3-MA (10 mM) significantly decreased LC3-II/I ratio and the expression of Atg5 (Figure 4A), accompanying with reduced the accumulation of GFP-LC3B puncta (Figure 4B). Importantly, autophagy inhibition by 3-MA rescued both HCT116 and HT29 cells from Sal B-induced cell death, which was demonstrated by flow cytometric analysis (Figure 4C).

To further explore the link between the inhibition of autophagy and Sal B-induced cell death, we silenced the expression of Atg 5, a key regulator of autophagy, with shRNA in HCT116 and HT29 cells. As shown in Figure 4D, the mRNA level of Atg5 was significantly downregulated, together with reduced the accumulation of GFP-LC3B puncta and decreased protein expression level of Atg5 (Figure 4B and 4E). To obtain objective quantification of cell death, we performed an Annexin V–FITC dual staining assay followed by flow cytometry. In agreement with the results from 3-MA, pretreatment with shAtg5 resulted in inhibiting Sal B-induced cell death (Figure 4F). In conclusion, induction of autophagy promotes the cell death of HCT116 and HT29 cells in response to Sal B, and inhibition of autophagy counteracts the cytotoxic effect of Sal B in these cells.

### AKT pathway is a critical mediator in regulating sal B-induced cell death

**Figure 5:**
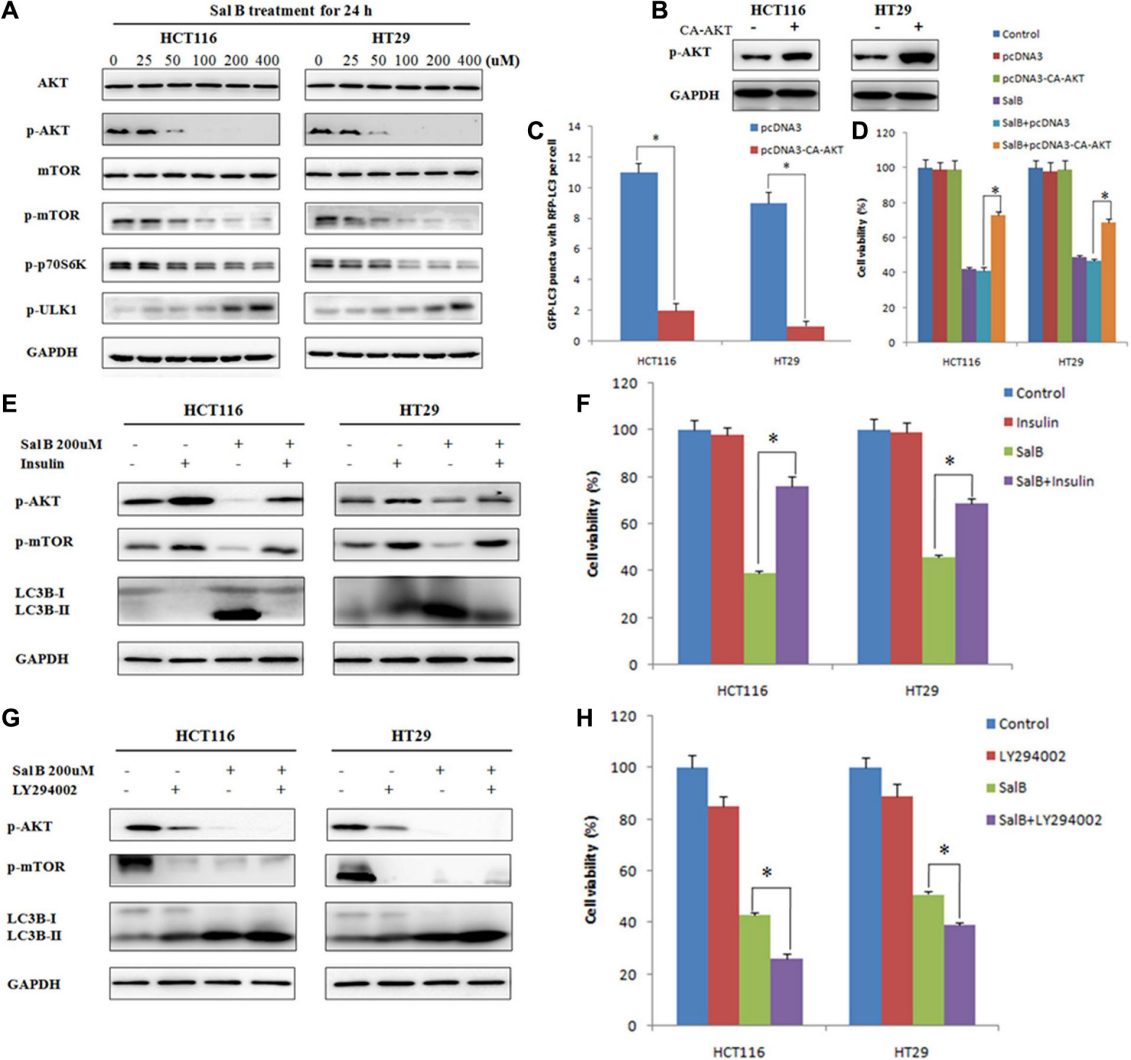
AKT pathway is a critical mediator in regulating sal B-induced cell death (**A**) The proteins associated Akt/mTOR axis were analyzed by western blotting after the cells were treated with Sal B. (**B**) HCT116 and HT29 cells were transiently transfected with pcDNA3-CA-AKT plasmid. (**C**) Cells were treated with Sal B in the presence or absence of pcDNA3-CA-AKT plasmid for 24 h before they were labeled with a fluorescent marker and imaged by fluorescence microscopy. Green: FITC-labeled LC3; Red: lyso-tracker-labeled lysosome; Blue: DAPI-labeled nucleus. *p < 0.05. (**D**) After transient overexpression of constitutively active AKT, cells were treated with Sal B for 24 h, and then cell viability was determined by MTT. (**E**) After pretreatment with insulin for 30 min, cells were treated with Sal B for 24 h, and then the expression of p-AKT, p-mTOR and LC3-II/I ratio were detected by western blotting. (**F**) After pretreatment with insulin for 30 min, cells were treated with Sal B for 24 h, and then cell viability was determined by MTT. *p < 0.05. (**G**) After pretreatment with LY294002, cells were treated with Sal B for 24 h, and then the expression of p-AKT, p-mTOR and LC3-II/I ratio were detected by western blotting. (**H**) After pretreatment with LY294002, cells were treated with Sal B for 24 h, and then cell viability was determined by MTT. *p < 0.05.

Previous studies have shown that Akt/mTOR axis plays an important role in the inhibition of autophagy [18, 19]. Therefore, we investigated whether the Akt/mTOR pathway was involved in Sal B-induced autophagic cell death in HCT116 and HT29 cells. Western blotting analysis showed that the phosphorylated levels of AKT, mTOR and p70 ribosomal protein S6 kinase (p70S6K) were significantly decreased but phosphorylated UNC-51-Like kinase 1 (ULK1) was enhanced in Sal B-treated cells. This result indicates a potent inhibitory effect of Sal B on Akt/mTOR signaling (Figure 5A).

To further identify the role of AKT in Sal B-induced autophagy, HCT116 and HT29 cells were transiently transfected with pcDNA3-CA-AKT plasmid. As shown in Figure 5B and 5C, overexpression of AKT significantly reduced Sal B-induced autophagy. Importantly, enforced expression of AKT remarkably attenuated the cytotoxicity of Sal B on HCT116 and HT29 cells (Figure 5D). Moreover, pretreatment with insulin, an activator of PI3K/AKT/mTOR signaling pathway, significantly enhanced the phosphorylation of Akt but decreased Sal B-induced autophagy and cell death (Figure 5E and 5F). Inversely, pretreatment with LY294002, an inhibitor of PI3K/AKT/mTOR signaling pathway, markedly suppressed the phosphorylation of Akt but enhanced Sal B-induced autophagy and cell death (Figure 5G and 5H). Taken together, these data demonstrate that AKT/mTOR signaling is a critical mediator of Sal B-induced autophagic cell death, and AKT inhibition augments Sal B-induced cell death through inducing autophagy.

### Sal B exerts autophagy induction and antitumor efficacy *in vivo*

**Figure 6:**
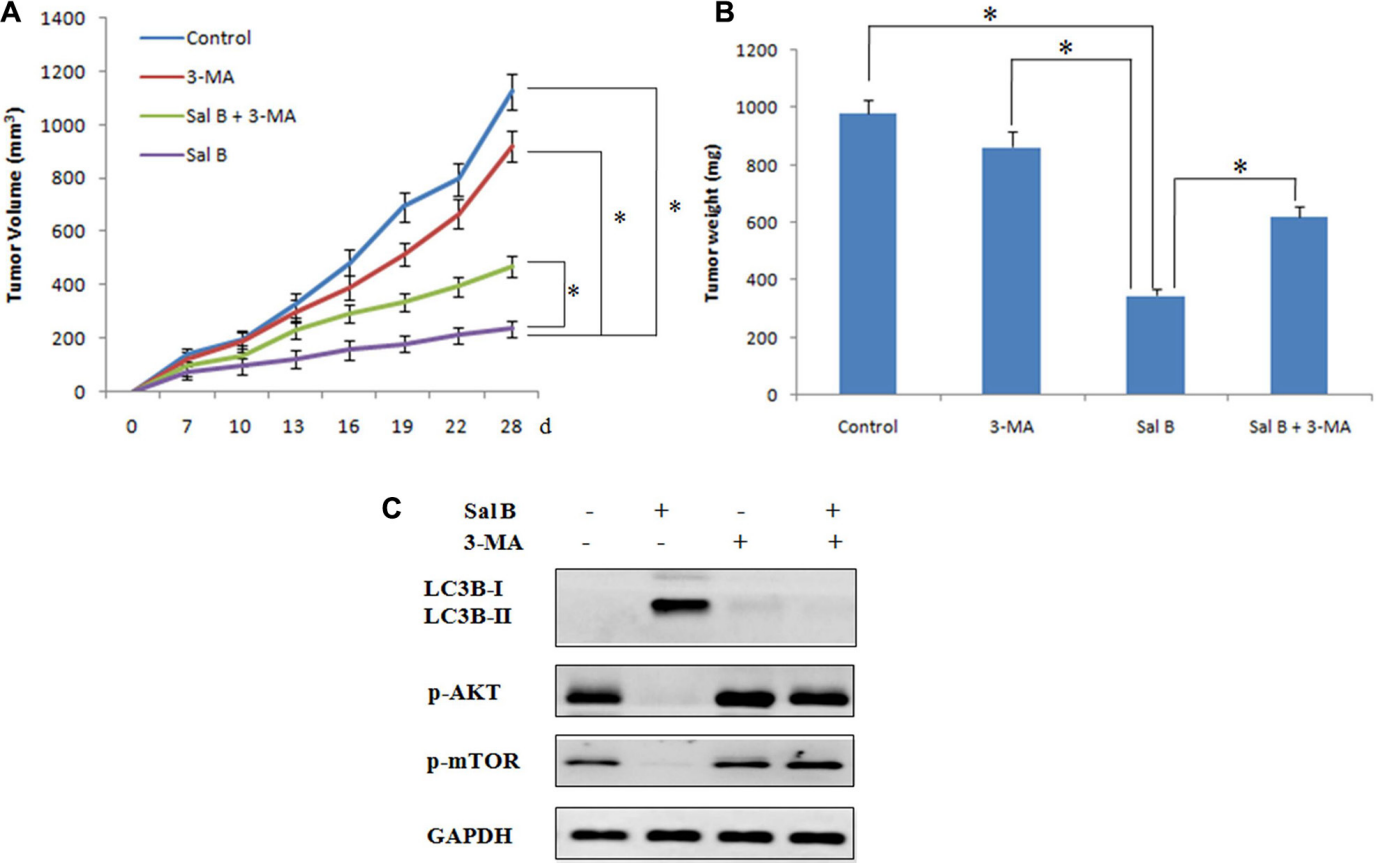
Sal B exerts autophagy induction and antitumor efficacy in HCT116 xenograft models (**A**) Tumor volume in each group. Data are expressed as the mean ± SEM. (**B**) Tumor weight in each group. Data were expressed as the mean ± SD, *p < 0.05. (**C**) Western blotting analysis of protein expression in tumors taken from mice.

To further determine the therapeutic potential of Sal B *in vivo*, we then injected BALB/c nude mice subcutaneously with HCT116 cells. The mice with tumor xenografts reaching 100 mm^3^ were randomly divided in to 4 experimental groups: control group, 3-MA group (20 mg/kg, intraperitoneal injection, every 4 days, for 28 days), Sal B group (80 mg/kg, intraperitoneal injection, once daily, for 28 days) and Sal B plus 3-MA group. As shown in Figure 6A and 6B, 3-MA alone had no significant effect on the growth of the tumors and Sal B alone displayed a strong antitumor activity. Consistent with the *in vitro* results, a combination with the autophagy inhibitor 3-MA significantly increased tumor growth compared with Sal B alone, indicating that Sal B also induced pro-death autophagy *in vivo*. Furthermore, no significant weight loss or hepatic toxicity was observed in the group of Sal B or a combination of 3-MA and Sal B (Figure did not show).

In order to investigate the mechanism underlying the inhibition of tumor growth by Sal B *in vivo*, we detected the expression of LC3B, p-AKT and p-mTOR by western blotting. As shown in Figure C, Sal B treatment resulted in an increase in LC3-II expression. Moreover, consistent with the *in vitro* results, Sal B induced autophagy through the inhibition of expression of p-AKT and p-mTOR (Figure 6C).

Taken together, our data suggest that Sal B inhibited tumor growth *in vivo* by inducing autophagy through suppressing AKT/mTOR pathway. Inhibition of autophagy by 3-MA potently attenuated the antitumor activity of Sal B *in vivo*.

## DISCUSSION

It is increasingly appreciated that targeting autophagy may be a promising new strategy for cancer drug discovery [20, 21]. Moreover, the natural herbal products that can inhibit or induce autophagy are attracting more and more attention in developing novel chemotherapeutics for cancers [22–24]. In the present study, we show that Sal B is a novel autophagy inducer and exerts its antitumor activity as a single agent in colorectal cancer cells. In the further study, we found that induction of autophagy may be a key mediator for Sal B-triggered cell death and inhibition of autophagy counteracted the cytotoxic effect of Sal B in HCT116 and HT29 cells. Finally, it was clearly suggested that Sal B induced autophagic cell death in CRC cell lines by the inhibition of AKT/mTOR signaling pathway.

Autophagy is regarded as a potential target for anticancer therapy and exerts complicated functions in different stages of cancer [25, 26]. Currently, many autophagy regulators have been identified as potential cancer therapeutic agents in pre-clinical and clinical studies [27]. Up till now, many natural products are also reported to trigger cancer cell death by regulating the signal pathways or functional status of autophagy [28, 29]. Sal B is a leading bioactive component of Danshen and has been widely used for thousands of years in oriental medicine to treat cardiovascular and cerebrovascular diseases [14]. Although previous study has shown that Sal B-induced autophagy may have the protective or lethal role in starving cardiac cells [30], autophagic cell death mediated by Sal B has never been recognized. In our study, Sal B was found to induce cell death and trigger autophagy in HCT116 and HT29 cells in a dose-dependent manner. We further examined the role of autophagy in Sal B-induced cell death via pretreatment with autophagy inhibitor or the silence of autophagy associated gene Atg5 with shRNA. Our results show that Sal B-induced cell death was rescued by autophagy inhibition, suggesting that Sal B-induced autophagy may play a pro-death role and contribute to the cell death of CRC cell lines.

AKT/mTOR signaling pathway has emerged as the central conduit in the regulation of autophagy. Accumulating evidence has highlighted that the inhibition of AKT and its downstream target mTOR and p70S6K contribute to the initiation of autophagy [31, 32]. Our results show that Sal B treatment exerts a inhibitory effect on AKT activation and the phosphorylation of its downstream targets, including mTOR and p70S6K. Overexpression of AKT by the transfection with pcDNA3-CA-AKT plasmid or pretreatment with insulin decreased Sal B-induced autophagy and cell death. Inversely, PI3K/AKT/mTOR signaling pathway inhibitor LY294002 treatment markedly enhanced Sal B-induced autophagy and cell death. Thus, AKT/mTOR signaling pathway is a critical mediator in regulating Sal B-induced cell death.

In conclusion, our present data show, for the first time, that Sal B is a novel autophagy inducer and exerts its antitumor activity as a single agent in colorectal cancer cells, both *in vitro* and *in vivo*, through the suppression of AKT/mTOR pathway. Our findings have important clinical implications. Next, we will compare the cytotoxicity of Sal B with or without combination of several anticancer drugs (such as 5-FU, Oxaliplatin and Irinotecan) in preclinical models and investigate the effect of Sal B on the invasion and metastasis of CRC cells.

## MATERIALS AND METHODS

### Reagents and antibodies

Sal B was purchased from the National Institute for the Control of Pharmaceutical and Biological Products (Beijing, China). BafA1 (B1793), 3-methyladenine (M9281), pan caspase inhibitor Z-VADFMK (C2105) were purchased from Sigma Aldrich. Proteins were reacted with one of the following: anti-LC-3B (#3868), anti-p62 (#8025), anti-Atg5 (#2630), anti-PARP (#9532), anti-cleaved PARP (#5625), anti-cleaved caspase-3 (#9661), anti-cleaved caspase-9 (#7237), anti-AKT (#9272), anti-phospho-AKT (Ser473) (#4060), anti-mTOR (#2972), anti-phospho-mTOR (Ser2448) (#5536), anti-phospho-p70S6K (Thr389) (#9234), anti- phospho-ULK1 (Ser757) (#6888) were purchased from Cell Signaling Technology.

### Cell lines and animals

Human colon cancer cell lines HCT116 and HT29 were obtained from American Type Culture Collection. HCT116 cells were maintained in RPMI 1640 medium (Invitrogen, 11875-093). HT29 cells were maintained in McCoy's 5A (Invitrogen, 16600-082). All media were supplemented with 10% (v/v) fetal bovine serum (FBS), 1% (v/v) of 100 U/ml penicillin and 100 mg/mlstreptomycin. Cells were cultured at 37°C in a humidified 5% CO_2_ incubator. Sal B was dissolved in double-distilled H_2_O and was further diluted with medium before use. Female athymic BALB/c nude mice (Shanghai Institute of Material Medicine, Chinese Academy of Science, China) were maintained in a specific pathogen-free facility and were treated with humane care after approval from the Animal Care and Use Committee of Zhejiang University.

### Measurement of cell viability and apoptosis

Cell viability was determined by MTT assay. Cells were seeded in 96-well flat bottom microtiter plates at a density of 1 × 10^4^ cells per well. 24 h later, Sal B was added at the concentrations indicated for 24 h. The absorbance was measured on a microplate reader (Synergy HT, Bio-Tek, USA) at 570 nm. Phartmingen annexin V-FITC Apoptosis Ddtection Kit I (BD, USA) was used to detect apoptosis and the estimation procedure was performed according to the manufacturer's instructions. 2 × 10^6^ cells were seeded into a 6 cm dish. After attachment overnight, cells were washed twice with PBS and the medium was replaced medium with Sal B for 24 h. All cells including the floating cells in the culture medium were harvested. The cells were resuspended in ice-cold 1 × binding buffer at a concentration of 1 × 10^6^ cells/ml. 100 μl of cell suspension were each mixed with 5 μl FITC Annexin V and 5 μl PI. The mixture was incubated for 15 min at room temperature in the dark and then analyzed by FACSCalibur Flow Cytometer (BD Biosystems, Heidelberg, Germany).

### Colony formation assays

Cells were trypsinized and plated in triplicate into 10 cm dishes at different densities based on cell types. Cells were treated with the indicated concentrations of Sal B or vehicle control for 24 h. Twenty-four hours after Sal B treatment, the medium was removed and cells were maintained in normal culturing medium. Two weeks after the cells were plated, they were washed and stained with crystal violet, and the colonies containing > 50 cells were counted.

### Semi-quantitative RT–PCR

Total RNA was isolated using TRIzol reagent (Invitrogen, CA, USA) according to the manufacturer's instructions. Approximately 2 μg of total RNA was converted to cDNA by First-strand cDNA synthesis kit (OriGene Technologies, MD, USA). Semi-quantitative RT–PCR was performed as described before. The GAPDH mRNA sequence was also amplified as an internal control.

### Immunofluorescent confocal laser microscopy for LC3 and lysosome co-location

Lysosome was firstly labeled by incubation with Lyso Tracker (Invitrogen, L7528), a lysosome reporter dye, for 90 min at 37°C. Cells were collected, fixed and permeabilized with 1% CHAPS buffer (150 mM NaCl, 10 mM HEPES, 1.0% CHAPS) at room temperature for 10 min, incubated with anti-LC3 for 2 h at room temperature, and washed with PBS, incubated for another 45 min.

### Plasmid and RNA interference

GPF-LC3 lentiviral plasmid (LTV-801) was purchased from Cell Biolabs, INC. Cells were transfected with either nonspecific siRNA (Qiagen,1027280) and Atg5 siRNA (Qiagen, SI02655310) via LipofectAMINE RNAi max (Invitrogen, 13778150) according to the manufacturer's instructions.

### Transmission electron microscopy

Treated cells were washed and fixed for 30 min in 2.5% glutaraldehyde. The samples were treated with 1.5% osmium tetroxide, dehydrated with acetone and embedded in Durcupan resin. Thin sections were poststained with lead citrate andexamined in the TECNAI 10 electron microscope (Philips, Holland) at 60 kV.

### Western blot analysis

Cells were harvested from cultured dishes and were lysed in a lysis buffer [20 mM Tris-HCl pH 7.6, 1 mM EDTA, 140 mM NaCl, 1% NP-40, 1% aprotinin, 1 mM phenylemethylsulfonyl fluoride (PMSF), 1 mM sodium vanadate]. Protein concentration was determined using a BCA Protein Assay Kit (Pierce). Cell lysates (40 μg protein/line) were separated on a 5 to 20% Tris-Tricine Ready Gel SDS-PAGE (Bio-Rad) for nitrocellulose membrane blotting. The blotted membranes were blocked with 5% skim milk for 1 h and were incubated with primary antibodies. The immunoreactive bands were visualized by enhanced chemiluminescence using horseradish perox-idase-conjugated IgG secondary antibodies. Band density was measured by densitometry, quantified using gel plotting macros of NIH image 1.62, and normalized to an indicated sample in the identical membrane.

### *In vivo* subcutaneous tumor model

All of the *in vivo* experimental protocols were approved by the animal care committee of Sir Run Run Shaw Hospital, Zhejiang University. Viable HCT116 cells (1 × 10^7^cells in 0.1 ml phosphate buffer saline) were injected subcutaneously into right dorsal flank of 6-week-old female BALB/c nude mice (six mice per group). Tumor volume was assessed every 2 days for 4 weeks. Tumor volume was calculated by the following formula: (short diameter)^2^ × (long diameter)/2.

### Statistical analyses

Results are expressed as values of mean ± standard deviation (SD). Statistical analysis was performed using SPSS 16.0 for Windows (SPSS Inc., Chicago, IL, USA). The correlation coefficient of two factors was evaluated using Chi-square and Fisher's exact tests. The survival of patients with colorectal adenocarcinomas was compared using the Kaplan–Meier method, and differences between the survival curves were tested using the logrank test. A *P-value* less than 0.05 is considered significant.
